# Local Anæsthesia

**Published:** 1866-11

**Authors:** Henry T. Godfrey

**Affiliations:** Benton, Wisconsin


*Local Anæsthesia.* By HENRY T. GODFREY, M. D., Benton, Wisconsin.

My apology for presenting the following cases to your notice is, not that they contain anything of novelty, but because they go to show the utility of Dr. Richardson's method of producing local anæsthesia to the country surgeon, who often finds it difficult or impossible to obtain the assistance necessary for the safe administration of chloroform. Sulphuric ether was used in the following cases:

1. Alice R——, æt. 16. Application of spray thirty seconds. Extraction of molar tooth, without pain. No ill effects.

2. Henry B——, æt. 12. Deep incised wound in back, caused by point of a plough. Application of spray forty seconds. Introduced three sutures, without pain. Wound healed well.

3. Same patient. Incised scalp wound. Application of spray thirty seconds. Introduced two fine sutures. Slight pain.

4. John S——, æt. 27. Onychia of three months' standing, which is so irritable that the slightest touch gives pain. Application of spray sixty seconds. Excision of one-fourth of nail, without pain.

5. Lawrence D——, æt. 60. Application of vapor thirty seconds. Extraction of molar tooth, without pain. This patient was under treatment for pneumonia, of which he died on the fourth day following.

6. Mrs. R——, æt. 30. Potts' fracture, followed by abscess of ankle-joint. This patient was so intractable during the early part of her illness, that she could not be induced to lie still, or permit any appliance to maintain the foot in proper position. Application of spray sixty seconds. Enlarged the wound. Extracted a piece of carious bone, without pain.

On the day following, opening of abscess, also, without pain, or other ill effect.

7. Mary Ann S——, æt. 17. Encysted tumor, size of a walnut, in the left superciliary region. Application of spray seventy seconds. Excision of tumor, without pain or ill effect.

